# Monoallelic and biallelic deletions of 13q14 in a group of CLL/SLL patients investigated by CGH Haematological Cancer and SNP array (8x60K)

**DOI:** 10.1186/s13039-015-0212-x

**Published:** 2016-01-06

**Authors:** Beata Grygalewicz, Renata Woroniecka, Jolanta Rygier, Klaudia Borkowska, Iwona Rzepecka, Martyna Łukasik, Agnieszka Budziłowska, Grzegorz Rymkiewicz, Katarzyna Błachnio, Beata Nowakowska, Magdalena Bartnik, Monika Gos, Barbara Pieńkowska-Grela

**Affiliations:** Cancer Genetic Laboratory, Pathology and Laboratory Diagnostics Department, Centre of Oncology, M. Skłodowska-Curie Memorial Institute, Warsaw, Poland; Flow Cytometry Laboratory, Pathology and Laboratory Diagnostics Department, Centre of Oncology, M. Skłodowska-Curie Memorial Institute, Warsaw, Poland; Department of Medical Genetics, Mother and Child Institute, Warsaw, Poland; Department of Pathology and Laboratory Diagnostics, Maria Skłodowska-Curie Memorial Cancer Center and Institute of Oncology, 15B Wawelska Str, 02-034, Warsaw, Poland

**Keywords:** CLL/SLL, 13q14 deletion, CGH and SNP array, UPD

## Abstract

**Background:**

Deletion of 13q14 is the most common cytogenetic change in chronic lymphocytic leukemia/small lymphocytic lymphoma (CLL/SLL) and is detected in about 50 % of patients by fluorescence in situ hybridization (FISH), which can reveal presence of del(13)(q14) and mono- or biallelic deletion status without information about the size of the lost region. Array-comparative genomic hybridization (aCGH) and single nucleotide polymorphism (SNP) can detect submicroscopic copy number changes, loss of heterozygosity (LOH) and uniparental disomy (UPD) regions. The purpose of this study was detection of the size of del(13)(q14) deletion in our group of patients, comparing the size of the monoallelic and biallelic deletions, detection of LOH and UPD regions.

**Results:**

We have investigated 40 CLL/SLL patients by karyotype, FISH and CGH and SNP array. Mutational status was of immunoglobulin heavy-chain variable-region *(IGVH)* was also examined. The size of deletion ranged from 348,12 Kb to 38.97 Mb. Detected minimal deleted region comprised genes: *TRIM13*, miR-3613, *KCNRG, DLEU2,* miR-16-1, miR-15a, *DLEU1*. The *RB1* deletions were detected in 41 % of cases. The average size in monoallelic 13q14 deletion group was 7,2 Mb while in biallelic group was 4,8 Mb. In two cases 13q14 deletions were located in the bigger UPD regions.

**Conclusions:**

Our results indicate that bigger deletion including *RB1* or presence of biallelic 13q14 deletion is not sufficient to be considered as adverse prognostic factor in CLL/SLL. CytoSure Haematological Cancer and SNP array (8x60k) can precisely detect recurrent copy number changes with known prognostic significance in CLL/SLL as well as other chromosomal imbalances. The big advantage of this array is simultaneous detection of LOH and UPD regions during the same test.

## Background

CLL/SLL is the most common leukemia in adults in Western countries [1]. The clinical course of this disease is very variable from indolent disease which is stable for many years to very rapid progression toward advanced stages, intensive treatment and short patients survival [2, 3]. Clinical staging systems developed by Rai and Binet can recognize advanced stage of disease, but they cannot predict disease course of the earlier stages [4]. Several prognostic markers have been described. Among genetic factors, prognostic significance have mutational status of *IGVH* and recurrent cytogenetic abnormalities [5, 6]. Somatic hypermutation of the *IGVH* gene is observed in approximately 50 % of patients, and its presence is associated with a more benign clinical course. Chromosomal changes of prognostic value as del(13)(q14), tris12, del(11)(q22.3) and del(17)(p13) can be detected in up to 50 % of patients by conventional cytogenetic analysis and up to 80 % by routine FISH analysis [7].

Deletion of 13q14 is the most common cytogenetic change in CLL/SLL and is detected in about 50 % of patients by FISH [5, 8]. This is a good prognostic factor if is detected as a sole aberration in FISH analysis. In karyotype del(13)(q14) is visible only in 8-10 % of patients, because in most of cases deletion size is submicroscopic [9]. Deletions vary considerably in size. The breakpoints are heterogeneous ranging from only 300 Kb up to more than 70 Mb [10–12]. The minimal deleted region (MDR) is described as located distal to *RB1* and comprises leukemia 2 (*DLEU2*) gene, which includes microRNA miR-15a/16-1 cluster [13–15]. In recent studies two main types of 13q14 deletions are proposed: del(13)(q14) type I (short), which breaks close to the miR16/15a locus and does not involve *RB1*; and del(13q)(q14) type II (larger), which includes *RB1* and has been suggested to be associated with greater genomic complexity and a more aggressive course [11, 16, 17]. Additionally 13q14 deletions may be heterozygous (monoallelic) or homozygous (biallelic). Studies of serial samples suggest that heterozygous deletion is an early event, whereas deletion of the second copy of this region occurs at a later stage [18, 19]. Biallelic del(13)(q14) are present in 30 % of 13q-deleted patients [20]. They are described as smaller and not involving *RB1* [11]. The large 13q deletions are most often monoallelic, whereas a minor proportion carries biallelic deletions. The 13q14 MDR includes miR-15a and miR-16-1, which are described as negative regulators of the *BCL2* expression [21]*.* One of the documented biological functions of miR-15a and 16–1 is down-regulation of the anti-apoptotic *BCL2* through post-translational mRNA repression, which may lead to an increased anti-apoptotic resistance [22]. This deletion allows the CLL/SLL cells to survive. Mouse models have formally proven the pathogenetic role of del(13q)(14) in CLL/SLL development. Three different lines of transgenic mice designed to mimic del(13q)(14) developed CLL/SLL and other del(13)(q14)-associated lymphoproliferative disorders [21, 23].

Array-based genomic technologies allow a genome wide screening for genetic lesions. An aCGH array enables the detection of acquired genomic copy number variations (CNV), excluding balanced chromosomal translocations. SNP array allows to detect the presence of deletions which are visible as a LOH regions and regions of copy-neutral LOH, which are also called uniparental disomies. The resolution of array is much higher than cytogenetic classical methods and enables detection of submicroscopic chromosomal changes. In the current study, we performed molecular analysis of 39 CLL/SLL patients using CytoSure Haematological Cancer and SNP array containing 60.000 probes. This array combines on one slide long oligo aCGH probes for copy number detection with SNP content for accurate identification of LOH also without concurrent changes in gene copy number. The aims of the current study were detection of the size of del(13)(q14) deletion in our group of CLL/SLL patients, comparing the size of the monoallelic and biallelic deletions, detection of LOH and UPD regions.

## Results

### Patients

Detailed genetic examination was conducted on a group of 40 patients, who had a loss of 13q14 region in the tumor cells in FISH analysis. At that time of analysis 25 % of patients were treated and 75 % of patients remain without treatment. Characteristics of patients are given in Table 1. The median age at the time of diagnosis was 62 years (range 24–78). The 55 % of the patients were male.

**Table 1:** Clinical characteristics of 40 CLL/SLL patients

Case No.	Sex	Age at diagnosis (y)	Diagnosis	Binet stage at enrollment	Time to treatment (mo)	Overall survival (mo)	CD38 > 30 % (1) CD38 ≤ 30 % (0)
1.	M	55	CLL	C	13	70+	1
2.	M	59	CLL	C	2	54	1
3.	M	73	CLL	C	18	43+	0
4.	M	58	SLL	C	1	27	1
5.	F	75	CLL	B	nd	73	1
6.	F	69	CLL	A	84	95+	0
7.	F	61	CLL	A	7	141+	1
8.	M	47	CLL	A	36	80+	0
9.	M	63	CLL	C	36	70+	1
10.	M	47	CLL	B	30	76+	0
11.	M	47	CLL	A	36	72+	0
12.	M	51	CLL	A	36+	36+	0
13.	M	34	CLL	A	20	139	0
14.	M	57	SLL	A	3	71+	1
15.	K	54	CLL	A	nt	138+	0
16.	M	67	CLL	C	1	17+	1
17.	M	24	CLL	C	64	148	1
18.	M	52	CLL	B	28	83	0
19.	F	63	SLL	A	76	96+	0
20.	M	64	CLL	C	4	42	1
21.	M	41	CLL	C	3	31+	1
22.	F	74	CLL	A	78	111	1
23.	F	63	CLL	B	53	62+	1
24.	M	78	CLL	C	7	32+	0
25.	M	64	CLL	C	0	66+	0
26.	F	51	CLL	A	nt	59+	0
27.	F	60	CLL	A	nt	175+	0
28.	F	66	CLL	A	nt	16+	0
29.	F	76	CLL	A	nt	8+	0
30.	M	76	CLL	C	30	48	1
31.	F	67	CLL	B	21	187+	0
32.	F	51	SLL	B	60	144+	0
33.	F	64	CLL	B	23	40+	1
34.	M	75	CLL	A	nt	39+	0
35.	F	64	CLL	B	89	107+	0
36.	M	76	CLL	C	0	5	0
37.	F	66	CLL	A	nt	59+	nd
38.	F	56	CLL	C	nt	131+	0
39.	M	53	CLL	C	1	8+	0
40.	F	60	CLL	C	0	39	nd

*y* years, *mo* months, *nd* no data, *nt* not treated

### Conventional G-banding analysis

Among the 40 examined patients the karyotype analysis was successful in 35 of cases (Table 2). In 12 of patients the karyotype was normal and 23 of patients showed non-random karyotype aberrations. Deletion of 13q14 was karyotypically visible in two patients (cases 3,12), monosomy 13 in one case (case 39) while translocations with 13q14 break point were noticed twice, as t(9;13)(q34;q14) and t(2;13)(q37;q14) (case 30 and 33). Six patients showed deletion of 11q, three presented trisomy 12, one patient displayed deletion of 17p as t(17;18)(p11.2;q11.2) translocation. Other changes had random occurrence.

**Table 2:** Results of karyotype analysis, FISH and *IGVH* mutation status of 40 CLL patients

Case no.	Karyotype	FISH analysis				Mutational status *IGVH*
		del 13q14	tris 12	del *ATM*	del *TP53*	
Cases with monoallelic deletion 13q14						
1.	46,XY[10]	97 %	N	N	N	UM
2.	-	97 %	N	88 %	N	UM
3.	46,XY,del(13)(q14q32)[2]/46,XY[17]	96 %	N	N	N	UM
4.	45,XY,-6,-13,+mar [6]/ 46,XY [1]	95 %	N	N	N	UM
5.	46,XX,del(11)(q21)[10]	95 %	N	99 %	N	UM
6.	46,XX[15]	94 %	N	N	N	UM
7.	46,XX,t(?;14)(?;q32),?add(18)(q23)[3]/46,XX[7]	93 %	N	N	N	M
8.	46,XY[12]	90 %	N	92 %	N	UM
9.	46,XY[20]/45 ~ 46,XY,-10[2], +1 ~ 3mar[cp3]	90 %	N	92 %	N	UM
10.	-	86 %	N	N	N	M
11.	47,XY,+?2,-8,+mar[3]/46,XY[37]	84 %	N	N	N	UM
12.	46,XY,del(13)(q14q14)[9]/46,XY[11]	80 %	N	N	N	UM
13.	-	71 %	N	N	N	M
14.	46,XY,del(11)(q21q24)[3]/46,XY[4]	71 %	N	81 %	N	UM
15.	46,XX[38]	69 %	N	N	N	M
16.	46,XY,del(11)(q23)[7]/46,XY,-13,+mar[7]/ 46,XY[4]	63 %	N	20 %	N	UM
17.	46,XY,add(1)(q?44),del(11)(q?14) [2]/46,XY [18]	58 %	N	38 %	N	UM
18.	47,XY,+12[1]/46,XY[10]	57 %	N	N	N	UM
19.	46,XX[20]	55 %	N	N	N	UM
20.	46,XY[19]	50 %	N	N	N	UM
21.	46,XY[19]	43 %	N	N	N	UM
**Cases with biallelic deletion 13q14**						
22.	-	**98 %**	N	N	39 %	UM
23.	46,XX,del(11)(q14)[10]	**90 %**	N	90 %	N	UM
24.	47,XY,-6,del(12)(p11.2),+del(12)(p11.2), +der(?) (?- > ?cen- > ?::6p25- > 6q21:6q14- > 6qter)[10] /46,XY[2]	**89 %**	76 %	N	N	M
25.	-	**89 %**	N	N	94 %	UM
26.	46,XX[20]	**87 %**	N	N	N	M
27.	46,XX,t(2;7)(p11;q22)[6]/46,XX[7]	**86 %**	N	N	N	M
28.	45,X,-X[6]/46,XX[14]	**80 %**	N	N	N	M
29.	47,XX,+12[9]/46,XX[1]	**56 %**	41 %	N	N	M
30.	46,XY,t(9;13)(q34;q14)[17]/46,XY[1]	**90 %** 10 %	N	N	N	UM
31.	46,XX[20]	**67 %** 19 %	N	N	N	M
32.	46,XX,+12,[6]/46,XX[5]	**44 %** 32 %	80 %	N	N	M
33.	46,XX,t(2;13)(q37;q14)[6]/46,XX[3]	**40 %** 53 %	N	N	N	UM
34.	46,XY[13]	**40 %** 40 %	N	N	N	M
35.	44 ~ 47,XX,+12[7]/46,XX[5]	**35 %** 7 %	74 %	N	N	M
36.	45,XY,der(17)t(17;18)(p11.2;q11.2),-18[2]/ 45,idem,-11,+mar[6]	**26 %** 70 %	N	27 %	93 %	UM
37.	46,XX[20]	**23 %** 21 %	N	N	N	M
38.	46,XX,del(5)(p11.2)[2]/46,XX[14]	**20 %** 62 %	N	N	N	M
39.	46,XY,del(11)(q21)[7]/45,idem,-13 [4]/46,XY[4]	**19 %** 76 %	N	85 %	N	UM
40.	46,XX[5]	**11 %** 86 %	N	95 %	N	UM

„-‘’ no katryotype, *del* deletion, *tris* trisomy, *N* 100 % cells with two normal copies, *M* mutated *IGVH*, *UM* unmutated *IGVH*, bold type 13q14 biallelic deletion clone

### FISH analysis

In 40 CLL/SLL cases with the presence of 13q14 deletion detailed analysis showed 21 of patients with monoallielic and 19 of patients with biallelic deletion. FISH results are shown in Table 2. Monoallelic deletion was present in the range of 43–97 % of cells (average 77.8 %) in individual cases. Biallelic deletion accounted 56–98 % (average 84.4 %) of cells population in separate cases. Pure biallelic deletion was observed in 8 patients (42 %) and accounted 56–98 % (average 84,4 %) of cells. The next 11 patients (58 %) had separate clones with combined monoallelic and biallelic 13q14 deletions (cases 30–40). Biallelic deletion clones were detected in 19–90 % of interphase nuclei (average 37.7 %) and monoallelic deletion clones were present in the range of 7–85 % of cells (average 43.3 %) in distinct cases. Other FISH changes were visible in 18/40 cases. The deletion of *ATM* was shown in 7 out of 21 monoallelic cases and in 4 out of 19 biallelic cases. Trisomy 12 (4 cases) as well as *TP53* deletion (3 cases) were seen only in biallelic group.

### *IGVH* mutational status

Analysis of the mutational status of *IGVH* in all 40 patients indicated 62 % of patients with unmutated (UM) and 38 % of patients with mutated (M) *IGVH* (Table 2). In monoallelic 13q14 deletion group UM status showed 81 % of patients while mutation of *IGVH* was detected in 19 % of patients. All 7 patients with *ATM* deletion in this group had UM *IGVH*. In biallelic 13q14 deletion group 58 % of patients revealed mutated *IGVH* status and 42 % unmutated status. All three patients with *TP53* deletion and four patients with *ATM* deletion showed UM *IGVH*, on the contrary all four patients with trisomy 12 had mutated *IGVH*.

### aCGH analysis

**Fig. 1:**
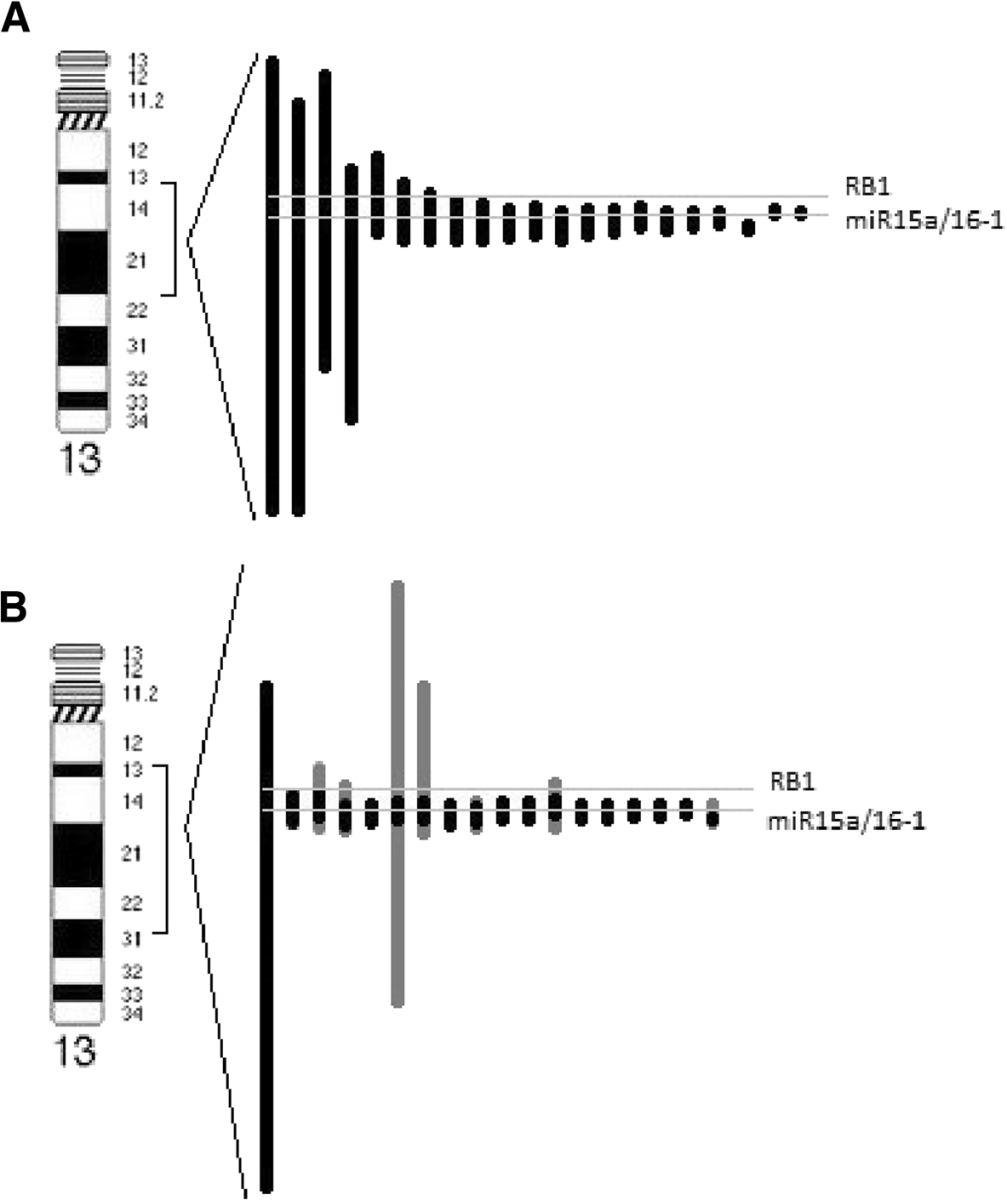
Pattern of chromosome 13q deletions of 39 CLL/SLL patients detected by CGH array. **a** monoallelic deletions (black lines); **b** biallelic deletion (grey lines indicate deletion size on the second chromosome 13 copy, if was different than on the first copy identified in array analysis)

CGH array analysis was performed on 39 available from 40 studied cases. Analysis confirmed 13q14 deletion in all patients (Table 3, Fig. 1). The size of deletion ranged from 348,12 Kb to 38.97 Mb. In all cases except one (case 14) deleted region contained miR-16-1 (position 50,623,109–50,623,197) and miR-15a (position 50,623,255–50,623,337) genes (Fig. 1a). The deletions including *RB1* were detected in 41 % of cases. In all 21 monoallelic cases the loss of 13q14 was detected as a single region. The average size in monoallelic 13q14 deletion group was 7,2 Mb. The smallest monoallelic MDR of 13q14 was 348,12 Kb and comprised genes: *TRIM13*, miR-3613, *KCNRG, DLEU2*, miR-16-1, miR-15a, *DLEU1.* The size of the biggest monoallelic deletion was 34,82 Mb. In case 14 monoallelic deletion of 13q14 not included miR-16-1 and miR-15a and contained fragment of *DLEU2, DLEU1, DLEU7*. The deletion proximal breakpoint was located 25,1 Kb telomeric direction from miR-16-1 and 24,9 Kb from miR-15a. The deletions including RB1 were detected in 9/21 (43 %) of monoalelic cases. Among 18 biallelic cases the same region of deletion on the both copies of chromosome 13 was identified in 11 (61 %) cases, while in next 7 patients (39 %) two different deleted regions were detected. The median size of 13q14 deletion in biallelic group was 4,8 Mb. The size of the MDR was 505,17 Kb. The biggest lost region was 38,97 Mb. All cases showed deletion of miR-16-1 and miR-15a. Deletion *RB1* was identified in 7/18 (39 %) of biallelic cases. Part of cytogenetic changes, detected by array CGH, confirmed presence of typical chromosomal aberrations identified by FISH (Table 4). Deletion of 11q was identified in 8 of 11 patients with *ATM* deletion detected by FISH. The smallest deletion del(11)(q22.1q23.3) was 16,96 Mb and the biggest del(11)(q14.1q25) covered 50,41 Mb. In six patients 11q deletion was interstitial whereas in other two cases (17,39) deletion was terminal. Trisomy 12 was identified in 4 patients (cases 24,29,32,35). In three out of these cases array analysis showed typical trisomy 12, while one patient (case 24) showed partial trisomy covering whole long arm of chromosome 12. Deletion of 17p was detected in all three patients with one copy of *TP53* in FISH (cases 22,25,36). The smallest 17p deletion del(17)(p13.3p13.1) was 7,64 Mb and in the biggest del(17)(p13.3p11.2) comprising almost whole short arm of chromosome 17 was 21,08 Mb. Additional changes, with respect to those detected by FISH, were similar in both groups with monoallelic and biallelic deletion of 13q14. The most common aberrations included losses and gains of different regions of 1q (4 cases), gains of 2p (3 cases) and 19q13 (3 cases) as well as changes of Xq (3 cases). The minimal gained region on 2p16.1-p15 (case 21) was 3,23 Mb and covered genes: *FANCL, EIF3FP3, BCL11A, PAPOLG, REL, NONOP2, PUS10, PEX13, KIAA1841, AHSA2, USP34.* Rest of copy number alternations had random occurrence. Additional copy number aberrations, in relation to the already described, were detected in 12 patients with monoallelic group and in 10 patients in biallelic group, with total number of alternation equal 20 in each group.

**Table 3:** Results of chromosome 13 array CGH analysis of 39 CLL patients

Case No.	Position of 13q14 deletion		Size of deletion	miR 15a/16-1 deletion	*RB1* deletion
	Monoallelic				
5.	arr 13q14.2q14.3	(50,561,374-50,909,490)x1	348,12 Kb	+	-
18.	arr 13q14.2q14.3	(50,575,469-51,213,898)x1	638,43 Kb	+	-
14.	arr 13q14.2q14.3	(50,648,212-51,296,645)x1	648.43Kb	-	-
10.	arr 13q14.2q14.3	(50,532,206-51,502,524)x1	970,32 Kb	+	-
11.	arr 13q14.2q14.3	(50,506,929-51,502,525)x1	995,60 Kb	+	-
17.	arr 13q14.2q14.3	(49,975,238-51,581,258)x1	1,61 Mb	+	-
6.	arr 13q14.2q14.3	(50,547,426-52,293,661)x1	1.75 Mb	+	-
19.	arr 13q14.2q14.3	(49,667,023-51,766,748)x1	2,10 Mb	+	-
21.	arr 13q14.2q14.3	(49,466,784-51,789,968)x1	2,32 Mb	+	-
1.	arr 13q14.2q14.3	(48,796,715-51,126,898)x1	2,33 Mb	+	-
4.	arr 13q14.2q14.3	(49,643,767-52,415,185)x1	2,77 Mb	+	-
20.	arr 13q14.2q14.3	(48,852,953-52,024,641)x1	3,17 Mb	+	-
9.	arr 13q14.2q14.3	(48,476,853-51,937,417)x1	3,46 Mb	+	+
3.	arr 13q14.2q14.3	(48,229,933-51,827,408)x1	3,60 Mb	+	+
8.	arr 13q14.2q14.3	(48,875,709-52,722,490)x1	3,85 Mb	+	+
16.	arr 13q14.13q14.3	(47,067,473-52,293,661)x1	5,23 Mb	+	+
7.	arr 13q14.11q14.3	(44,820,708-51,472,821)x1	6.65 Mb	+	+
12.	arr 13q14.12q21.31	(45,230,434-65,085,253)x1	19,85 Mb	+	+
15.	arr 13q13.3q21.2	(37,178,772-60,025,895)x1	22,85 Mb	+	+
2.	arr 13q13.3q21.33	(39,377,596-71,248,873)x1	31,87 Mb	+	+
13.	arr 13q13.3q21.33	(36,430,114-71,248,873)x1	34,82 Mb	+	+
	Biallelic				
33.	arr13q14.2q14.3	(50,659,348-51,164,513)x0	505,17Kb	-	-
	arr 13q14.2q14.3	(50,337,728-51,897,968)x0	1,56 Mb	+	-
38.	arr 13q14.2q14.3	(50,575,469-51,360,705)x0	785,24 Kb	+	-
25.	arr 13q14.2q14.3	(50,597,418-51,454,330)x0	856.91Kb	+	-
27.	arr 13q14.2q14.3	(50,575,469-51,441,414)x1	865,95 Kb	+	-
24.	arr 13q14.2q14.3	(50,561,374-51,441,414)x0	880,04 Kb	+	-
26.	arr 13q14.2q14.3	(50,575,469-51,472,821)x0	897,35 Kb	+	-
35.	arr 13q14.2q14.3	(50,575,469-51,502,524)x1	927,06 Kb	+	-
	arr 13q14.2q14.3	(48,754,460-52,536,626)x1	3,78 Mb	+	+
29.	arr 13q14.2q14.3	(50,575,469-51,523,591)x1	948,12 Kb	+	-
37.	arr 13q14.2q14.3	(50,532,206-51,502,524)x1	970,32 Kb	+	-
28.	arr 13q14.2q14.3	(50,484,540-51,524,424)x1	1,04 Mb	+	-
	arr 13q14.2q14.3	(50,241,416-51,524,424)x1	1,28 Mb	+	-
34.	arr 13q14.2q14.3	(50,484,540-51,572,737)x1	1.09 Mb	+	-
32.	arr 13q14.2q14.3	(50,408,714-51,572,737)x0	1,16 Mb	+	-
	arr 13q13.3q14.3	(39,596,989-51,624,965)x1	12,03 Mb	+	+
31.	arr 13q14.2q14.3	(50,305,714-51,469,354)x1	1,16 Mb	+	-
	arr 13q12.3q21.31	(31,346,665-64,680,548)x1	33,33 Mb	+	+
36.	arr 13q14.2q14.3	(49,579,386-51,404,793)x1	1,83 Mb	+	-
40.	arr 13q14.2q14.3	(49,667,023-51,641,879)x1	1,97 Mb	+	-
	arr 13q14.2q14.3	(48,783,721-52,722,490)x1	3,94 Mb	+	+
22.	arr 13q14.2q14.3	(48,841,955-50,976,908)x0	2,13 Mb	+	+
	arr 13q14.13q14.3	(46,934,009-52,663,754)x0	5,73 Mb	+	+
30.	arr13q14.2q14.3	(48,801,028-51,680,357)x0	2,88 Mb	+	+
39.	arr 13q14.11q31.1	(41,246,428-80,220,989)x1	38.97 Mb	+	+

In biallelic 13q14 deletion group digit x1 suggests monoallelic change, but this value is associated with lower percentage of cells with biallelic deletion in whole cell population

**Table 4:** Results of aCGH analysis of copy number variations, and SNP results revealing loss of heterozygosity, and uniparental disomy status of 39 CLL patients

Case no.	CNV	LOH	UPD
Cases with monoallelic deletion			
1.	arr 13q14.2q14.3(48,796,715-51,126,898)x1 (2,33 Mb)	n.t.	n.t.
2.	arr 11q13.4-q23.3(70,503,170-116,961,197)x1 (46,46 Mb)	11q14q22.3(81,735,918-104,024,380) (22,29 Mb)	No changes
	arr 13q13.3q21.33(39,377,596-71,248,873)x1 (31,87 Mb)	13q13.2 q22.1(33,900,810-74,999,739) (41,09 Mb)	
3.	arr 13q14.2q14.3(48,229,933-51,827,408)x1 (3,60 Mb)	No changes	No changes
	arr 19q13.41(52,273,095-52,540,512)x3 (267,42 Kb)		
4.	arr 13q14.2q14.3 (49,643,767-52,415,185)x1 (2,77 Mb)	No changes	No changes
5.	arr 4p15.2(25,475,860-26,940,881)x1 (1,47 Mb)		7q35q36.3(147,741,217-157,731,561)x2 (9,99 Mb)
	arr 11q14.3(89,656,697-91,983,518)x1 (2,33 Mb)		
	arr 11q21-q24.3(94,371,784-128,262,880)x1 (33,89 Mb)	11q22.1q24.3(99,501,357-129,280,824) (29,78 Mb)	
	arr 13q14.2q14.3(50,561,374-50,909,490)x1 (348,12Kb)		
	arr 17q21.31(44,204,228-44,418,272)x1 (214,04Kb)		
	arr Xp22.31(6,493,087-8,034,106)x3 (1,54 Mb)		
	arr Xq28(155,169,566-155,234,551)x3 (64,98 Mb)		
6.	arr 13q14.2q14.3(50,547,426-52,293,661)x1 (1.75 Mb)	No changes	No changes
7.	arr 13q14.11q14.3(44,820,708-51,472,821)x1 (6.65 Mb)	13q14.11q14.3(44,820,708-51,472,821)x1 (6.65 Mb)	No changes
8.	arr 11q22.1-q23.3(98,280,345-115,237,704)x1 (16,96 Mb)	11q22.1q23.3(97,723,221-115,807,318) (18,08 Mb)	No changes
	arr 13q14.2q14.3(48,875,709-52,722,490)x1 (3,85 Mb)		
9.	arr 2p25.3-p11.2(28,080,-84,775,088)x3 (84,75 Mb)		No changes
	arr 11q21-q23.3(93,076,720-116,285,664)x1 (23.21 Mb)	11q21q23.3(92,940,850-119,863,407) (26,92 Mb)	
	arr 13q14.2q14.3(48,476,853-51,937,417)x1 (3,46 Mb)		
	arr 17p13.3(10,152-1,130,849)x1 (1,12 Mb)		
10.	arr 13q14.2q14.3(50,532,206-51,502,524)x1 (970,32 Kb)	n.t.	n.t.
11.	arr 8q21.3-q24.3(89,582,111-143,980,245)x3 (54,4 Mb)	No changes	No changes
	arr 13q14.2q14.3(50,506,929-51,502,525)x1 (995,60Kb)		
12.	arr 13q14.12q21.31(45,230,434-65,085,253)x1 (19,85 Mb)	13q14.11q21.31(41,189,113-64,888,985) (23,70 Mb)	7q21.11q22.1(86,118,243-99,637,271) (13,52 Mb)
	arr 16q23.2 (79,630,721-79,634,651)x3 (3,93Kb)		
13.	arr 1q21.3-q22(154,947,320-155,300,504)x3 (353,18Kb)		No changes
	arr 13q13.3q21.33(36,430,114-71,248,873)x1 (34,82 Mb)	13q13.3q21.33(36,430,114-71,248,873) (34,82 Mb)	
14.	arr 11q14.1-q23.3(77,433,358-119,173,987)x1 (41,74 Mb)	11q13.1q23.3(64,336,971-115,680,986) (51,34 Mb)	No changes
	arr 13q14.2q14.3(50,648,212-51,296,645)x1 (648.43Kb)		
15.	arr 13q13.3q21.2 (37,178,772-60,025,895)x1 (22,85 Mb)	n.t.	n.t
16.	arr 13q14.11(41,500,387-42,681,278)x1 (1,18 Mb)	No changes	3p26.1p24.3 (6,131,168-18,306,025) (12,17 Mb)
	arr 13q14.13q14.3(47,067,473-52,293,661)x1 (5,23 Mb)		
17.	arr 8q24.23-q24.3(139,637,331-146,147,478)x3 (6,51 Mb)	n.t.	n.t
	arr 9p24.1(5,073,751-5,093,784)x3 (20,03Kb)		
	arr 11q14.1-q25(84,360,979-134,772,193)x1 (50,41 Mb)		
	arr 13q14.2q14.3(49,975,238-51,581,258)x1 (1,61 Mb)		
	arr Xq27.2(140,354,604-140,762,836)x0 (408,23Kb)		
18.	arr 6q15-q22.31(92,567,028-122,238,549)x1 (29,67 Mb)	n.t.	n.t.
	arr 13q14.2q14.3 (50,575,469-51,213,898)x1 (638,43Kb)		
19.	arr 3q23(140,617,291-142,215,033)x1 (1,6 Mb)	No changes	No changes
	arr 10q25.1(110,247,081-111,224,354)x3 (977,27Kb)		
	arr 13q14.2q14.3(49,667,023-51,766,748)x1 (2,10 Mb)		
20.	arr 13q14.2q14.3(48,852,953-52,024,641)x1 (3,17 Mb)	n.t.	n.t.
	arr 19q13.41(53,209,131-53,472,835)x3 (263,7Kb)		
21.	arr 2p16.1-p15(58,413,294-61,643,329)x3 (3,23 Mb)	No changes	No changes
	arr 13q14.2q14.3(49,466,784-51,789,968)x1 (2,32 Mb)		
Cases with biallelic deletion			
22.	arr 3p12.3-p11.1(77,467,782-90,191,784)x3 (12,72 Mb)	n.t.	n.t.
	arr 13q14.2q14.3(48,841,955-50,976,908)x0 (2,13 Mb)		
	arr 13q14.13q14.3(46,934,009-52,663,754)x0 (5,73 Mb)		
	arr 15q14-q15.1(40,067,479-42,229,801)x1 (2,16 Mb)		
	arr 17p13.3-p13.1(111,956-9,547,885)x1 (9,44 Mb)		
24.	arr 6p25.3(209,906-1,318,308)x3 (1,11 Mb)	n.t.	n.t
	arr 6p21.1-q13(45,388,754-72,422,581)x1 (27,03 Mb)		
	arr 12q11-q24.33(37,896,066-133,773,393)x3 (95,88 Mb)		
	arr 13q14.2q14.3(50,561,374-51,441,414)x0 (880,04Kb)		
25.^a^	arr 1q42.12q42.3(225,534,669-234,720,224)x1 (9.19 Mb)		
	arr 1q44(247,898,601-249,228,445)x3 (1.33 Mb)		
	arr 2p16.1p14(59,068,992-66,342,421)x3 (7.27 Mb)	2p16.1p14(59,068,992-66,342,421)x3 (7.27 Mb)	2p25.3p14(852,240-65,905,900) (65.05 Mb)
	arr 5q21.3 (108,305,821-108,763,974)x1 (458.5Kb)		
	arr 8p23.3p11.21(1,775,777-42,326,846)x1 (40.55 Mb)	8p23.3p11.1(591,022-43,149,647) (42.56 Mb)	
	arr 13q14.2q14.3(50,597,418-51,454,330)x0 (856.91Kb)	13q14.2q14.3(50,597,418-51,454,330)x0 (856.91Kb)	13q12.11q34(19,813,548-114,888,975) (95.08 Mb)
	arr 17p13.3p13.1(10,152-7,654,148)x1 (7.64 Mb)	17p13.3p13.1 (1,135,130-8,800,337) (7.67 Mb)	
	arr 17p12p11.2(11,403,886-21,438,821)x1 (10.03 Mb)	17p12p11.2 (11,724,886-21,047,102) (9.32 Mb)	
	arr 19q13.43(56,465,808-59,057,705)x3 (2.59 Mb)		
	arr Xq28(154,844,440-155,234,551)x2 (390.11Kb)		
26.	arr 7q34(142,034,557-142,424,354)x3 (389,8Kb)	No changes	No changes
	arr 13q14.2q14.3(50,575,469-51,472,821)x0 (897,35Kb)		
27.	arr 13q14.2q14.3(50,575,469-51,441,414)x1 (865,95Kb)		
	arr 17q21.31(44,204,228-44,342,442)x0 (138,21Kb)	17q21.31(44,204,228-44,342,442)x0 (138,21Kb)	17q21.2q21.33 (38,973,99-48,047,566) (9,07 Mb)
28.	arr 13q14.2q14.3(50,484,540-51,524,424)x1 (1,04 Mb)	No changes	No changes
	arr 13q14.2q14.3(50,241,416-51,524,424)x1 (1,28 Mb)		
29.			7q32.2q36.1(129,639,751-148,168,183) (18,53 Mb)
	arr 12p13.33-q24.33(207,344-133,773,393)x3 (133,57 Mb)		12q23.1q24.13(100,260,227-113,728,620) (13,49 Mb)
	arr 13q14.2q14.3(50,575,469-51,523,591)x1 (948,12Kb)	13q14.2q14.3(50,575,469-51,523,591)x1 (948,12Kb)	13q14.11q21.32(40,405,019-66,081,272) (25,68 Mb)
30.	arr13q14.2q14.3(48,801,028-51,680,357)x0 (2,88 Mb)	n.t.	n.t.
31.	arr 13q14.2q14.3(50,305,714-51,469,354)x1 (1,16 Mb)	n.t.	n.t
	arr 13q12.3q21.31(31,346,665-64,680,548)x1 (33,33 Mb)		
32.	arr 7q33-q34(134,286,767-143,042,219)x1 (8,76 Mb)	n.t.	n.t.
	arr 12p13.3-q24.33(207,344-133,773,393)x3 (133,57 Mb)		
	arr 13q14.2q14.3(50,408,714-51,572,737)x0 (1,16 Mb)		
	arr 13q13.3q14.3(39,596,989-51,624,965)x1 (12,03 Mb)		
33.	arr 2q37.3(238,903,162-242,335,337)x1 (3,43 Mb)	No changes	No changes
	arr 13q14.2q14.3(50,659,348-51,164,513)x0 (505,17Kb)		
	arr 13q14.2q14.3(50,337,728-51,897,968)x0 (1,56 Mb)		
34.	arr 13q14.2q14.3(50,484,540-51,572,737)x1 (1.09 Mb)	No changes	No changes
35.	arr 1q23.3(161,493,499-161,619,000)x3 (125,5Kb)	No changes	No changes
	arr 12p13.33-q24.33(151,196-133,773,393)x3 (133,62 Mb)		
	arr 13q14.2q14.3(50,575,469-51,502,524)x1 (927,06Kb)		
	arr 13q14.2q14.3(48,754,460-52,536,626)x1 (3,78 Mb)		
36.	arr 13q14.2q14.3(49,579,386-51,404,793)x1 (1,83 Mb)	n.t.	n.t.
	arr 17p13.3-p11.2(10,152,21-21,088,538)x1 (21,08 Mb)		
	arr 18p11.32-p11.21(2,857,465-14,096,343)x1 (11,24 Mb)		
37.	arr 13q14.2q14.3(50,532,206-51,502,524)x1 (970,32Kb)	n.t.	n.t.
38.	arr 13q14.2q14.3(50,575,469-51,360,705)x0 (785,24Kb)	No changes	No changes
39.	arr 1q43q44(240,340,273-249,228,445)x3 (8.89 Mb)		No changes
	arr 11q21q25(93,214,146-134,931,948)x1 (41.72 Mb)	11q21q25(92,940,850-133,599,968) (40.66 Mb)	
	arr 12p13.31p12.3(8,514,368-15,095,031)x1 (6.58 Mb)	12p13.32p12.3(3,782,056-18,007,840) (14.23 Mb)	
	arr 13q14.11q31.1(41,246,428-80,220,989)x1 (38.97 Mb)	13q14.11q22.1(41,189,113-73,649,362) (32.46 Mb)	
40.	arr 11q14.1-q24.2(83,780,266-127,200,577)x1 (43,42 Mb)	n.t.	n.t.
	arr 13q14.2q14.3(49,667,023-51,641,879)x1 (1,97 Mb)		
	arr 13q14.2q14.3(48,783,721-52,722,490)x1 (3,94 Mb)		

Changes are described in a cytogenetic region, molecular position and size in base pairs

*CNV* copy number variations, *LOH* loss of heterozygosity, *UPD* uniparental disomy, *n.t* not tested,^a^UPD of whole chromosome 13 with deletion 13q14

### SNP analysis

**Fig. 2:**
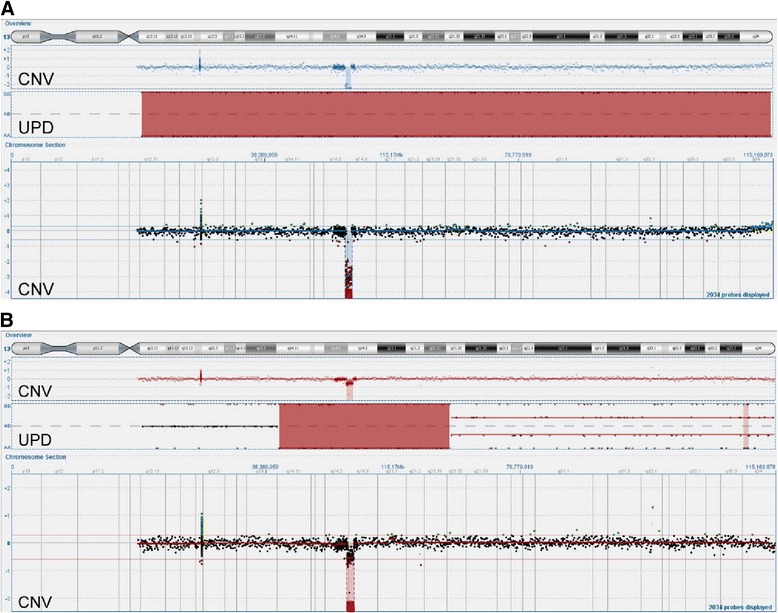
CytoSure Haematological Cancer and SNP array of two cases: 25 (**a**) and 29 (**b**). The overview window shows ideogram of chromosome 13, below result of aCGH as a copy number variations (CNV) indicating deletion of 13q14, underneath big red blocks demonstrating uniparental disomy (UPD) regions revealed in SNP analysis. Lower section shows magnification of aCGH analysis (CNV)

SNP analysis was performed on 25/39 cases of which 13/25 showed aberrant SNP pattern (Table 4). Chromosome 13 changes were detected in 7/25 patients. In five cases (2,7,12,13,39) SNP distribution confirmed big 13q14 deletions as LOH regions. In six cases (2,5,8,9,14,39) SNP analysis showed LOH in 11q deletion regions. In two patients (25,39) LOH regions matched deletions of 8p, 17p and 12p, respectively. Regions of no changes in copy number but with aberrant pattern in SNP analysis were considered as UPD. In two cases (25,29) 13q14 deletions were located in the bigger (at least 10 Mb larger than deletion regions) copy neutral LOH regions (Fig. 2). In case 25 this UPD covered whole chromosome 13. Remaining UPD regions included: 2p25.3-p14, 3p26.1-p24.3, 7q21.11-q22.1, 17q21.2-q21.33, 7q32.2-q36.6, 7q35-q36.3, 12q23.1-q24.13. In case 25 big UPD region (65 Mb) on 2p covered smaller deletion (7,27 Mb).

### Survival and time to treatment

**Fig. 3:**
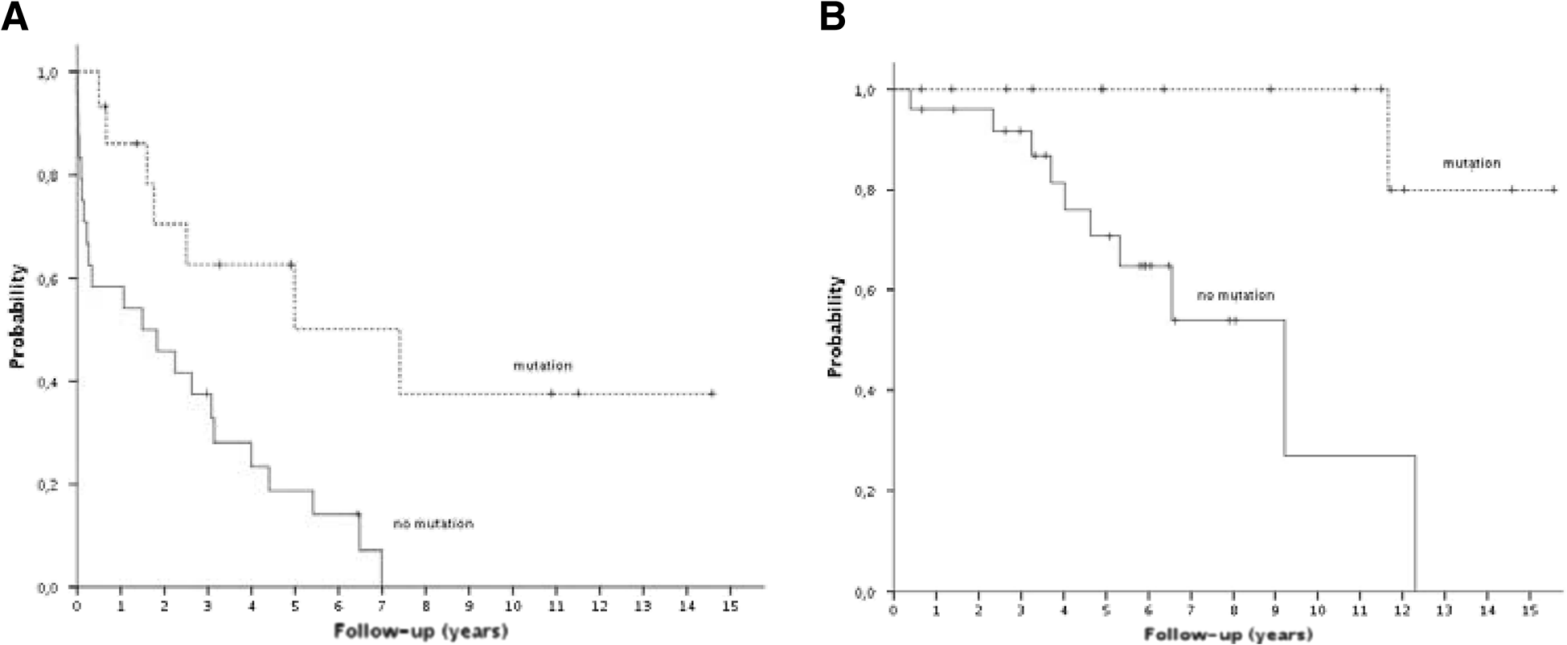
Statistical analysis of 40 CLL/SLL patients. **a**. Time to treatment patients with and without *IGVH* mutation (*P* = 0.003). **b** . Overall survival patients with and without *IGVH* mutation (*P* = 0.003)

Clinical follow-up of 40 CLL patients ranged from 8 to 187 months, with a median follow-up of 71 months. At the time of last follow-up 5 of 21 patients in monoallelic group and 4 of 19 patients in biallelic group had died. Time to treatment (TTT) for all patients ranged from 8 to 175 months, with a median TTT of 59 months. We investigated the relationship of 13q14 deletion status (monoallelic vs. biallelic; monoallelic vs. biallelic excluding cases with *TP53* and *ATM* deletion), size of 13q14 deletion (13q14 with *RB1* deletion vs.13q14 without *RB1* deletion) and *IGVH* mutation status with TTT and overall survival (OS) (Table 5). This analysis showed that only mutational status has statistically significant relation (Fig. 3). Median TTT was shorter in the unmutated group (18 months vs. 89 months, *P* = 0.003, 95 % CI: 0–45 and 16–162). Median OS was also shorter in *IGVH* unmutated group (110 months, *P* = 0.003; 95 % CI: 62–160) compared to the mutated group (median has not been reached).

**Table 5:** Statistical analysis of 40 CLL/SLL patients

Genetic feature		Number of patients		Median TTT (months)	Median OS (months)
		TTT	OS		
*IGHV* status	unmutated	24	25	18	110
	mutated	15	15	89	Not reached
	*P*-value			*p* = 0,003	*p* = 0,003
13q14 deletion	monoallelic	20	21	19	140
	biallelic	19	19	53	148
	*P*-value			*p* = 0,203	*p* = 0,511
13q14 deletion without del*TP53*	monoallelic	20	21	19	140
	biallelic	16	16	60	Not reached
	*P*-value			*p* = 0,099	*p* = 0,237
13q14 deletion without del *TP53* and del *ATM*	monoallelic	14	14	19	140
	biallelic	14	14	60	Not reached
	*P*- value			*p* = 0,141	*p* = 0,444
13q14 deletion	with *Rb* deletion	16	16	21	140
	without *Rb* deletion	23	24	30	148
	*P*- value			*p* = 0,426	*p* = 0,942

*TTT* time to treatment, *OS* overall survival

## Discussion

Only 8–10 % of 13q14 deletion can be detected in karyotype analysis in CLL/SLL patients because of its submicroscopic size [9]. By FISH method deletion of 13q14 is revealed in 50 % of patients. This technique can show the presence or absence of the deletion without information about the size of the lost region. Here we present detailed size analysis of 39 CLL/SLL patients performed by CytoSure Haematological Cancer and SNP array. The smallest identified 13q14 deleted region was 348,12 Kb. This observation is concordant with other studies, were MDRs were similar sizes and also comprised *DLEU1, DLEU2* and *DLEU7* genes [12, 24, 25]. In the most CLL/SLL cases 13q14 deletion leads to loss of two microRNA genes miR-15a and miR-16-1, which are considered to be a key genes of this deletion. Studies on the structure of genes in 13q14 deleted region revealed that in MDR is located *DLEU2* gene which encodes part of first exon of *DLEU1* as well as two microRNA miR-15a and miR-16-1 which are located between exons 2 and 5 of the *DLEU2* [26]. Previous data reported downregulation of miR-15a and miR-16-1 in about 65 % of CLL cases with 13q14 deletion [15]. However recent reports describe much smaller proportion of patients with downregulation of both micoRNAs which accounts near 10 % of CLL and mostly in patients with biallelic 13q14 deletion [11, 27, 28]. MiR-15a and miR-16-1 expression was inversely correlated to *BCL2* expression in CLL [22]. *BCL2* is an oncogene promoting survival by inhibiting cell death. In light of recent research that do not indicate a reduced expression of miR-15a and miR-16-1 in the majority of patients with 13q14 deletion but in the same time shows elevated level of *BCL2* protein in patients with monoallelic and biallelic 13q14 deletion, this point out that the regulation of BCL2 protein levels is more complex and do not mainly determined by miR-15a and miR-16-1 levels [28]. In our study one patient with 13q14 deletion detected by FISH retained both copies of miR-15a and miR-16-1. The proximal deletion breakpoint was situated telomeric direction relative to both microRNA genes. Similar phenomenon of 13q14 deletions without loss of miR-15a and miR-16-1 were described by Mosca et al. and Edelmann et al. [12, 24].

Deletion of the second copy of D13S319 locus in CLL/SLL is well documented. Biallelic 13q14 deletion can have the same or different sizes [16, 17, 24, 29]. Generally, biallelic deletions of 13q14 are reported as smaller in comparison with monoallelic deletions [10, 12, 24]. Our results indicate that biallelic 13q14 deletion regions can be the same or different sizes on both copies of chromosome 13. Concurrently the median size of deletion in biallelic group was much smaller than in monoallelic group what is consistent with the literature data. Some authors define biallelic 13q14 deletion presence as well as bigger deletion region covering *RB1* (called type II deletions) as adverse prognostic factors connected with faster lymphocyte growth and associated with inferior prognosis [11, 30, 31]. The statistical analysis of our data regarding to TTT and OS do not confirm this observations. Our data are in line with results of other groups, which showed that loss of second copy of 13q14 is not enough to cause a worst prognosis in CLL and there is not any significant difference in the baseline characteristic and TTT between patients with shorter (biallelic) and wider (monoallelic) 13q14 deletions [12, 25, 32, 33].

The presence of all cytogenetic aberrations identified by FISH was confirmed by aCGH. Only in three cases deletion 11q was not recognized in aCGH study. In two patients percentage of cells with *ATM* deletion was less than 30 % what was below the sensitivity of the method and one patient with del 11q was not analysed by aCGH. Among the most frequent additional changes revealed by aCGH the most significant was gain of 2p detected in three patients. This aberration is described as recurrent genetic change in CLL associated disease progression. Some studies defined in common 2p gained region presence of *REL, MYCN* and *ALK* oncogenes [34, 35]. The results of other research by Pfeifer and Edelmann delineated much smaller minimal 2p gained regions, which included 2p16 (size 3,5 Mb) and 2p16.1-p15 (size 1,9 Mb), respectively. Both regions contained two oncogenes *REL* and *BCL11A* [24, 30]. Our results are consistent with these observations. The size of minimal detected 2p16.1-p15 gained region was 3.23 Mb and included *REL* and *BCL11A* oncogenes. In second patient duplicated 2p16.1-p14 region was bigger and covered 7.27 Mb, consisting *REL* and *BCL11A*, but not *MYCN* and *ALK.* Third patient revealed duplication of the whole 2p. Additional copies of 2p in CLL are associated with unmutated *IGVH*, frequent occurrence of deletion 11q and 17p and advanced stage of disease [30, 34, 35]. In our studied group all three patients revealed unmutated *IGVH* and Binet stage C. One patients had deletion of *ATM* and other deletion of *TP53*. The presence of 2p gain often is accompanied by adverse genetic changes and more advanced stage of disease what confirms the poor prognosis of this change.

There is an association between prognosis and the somatic hypermutation status of the *IGHV* genes in CLL [5, 6]. Patients with unmutated *IGHV* display a more aggressive disease, high-risk cytogenetics and a poor outcome, while mutated *IGHV* are associated with a more favourable clinical course with long OS. In our analysed group all CLL/SLL patients with unfavourable cytogenetic prognostic factors as deletions of *TP53* and *ATM* had unmutated *IGVH* status, what confirms poor prognosis. On the contrary all patients with trisomy 12, which is associated with an intermediate prognosis and a good response to treatment, had mutated *IGVH*. Mutational status of *IGVH* was the only factor in our study with statistical significance in relation to TTT and OS. In both analysis patients with unmutated *IGVH* had shorter TTT and OS.

SNP array can identify LOH regions as well as copy neutral LOH, which are also called UPD in cancer genome. These chromosomal regions are characterized by loss of heterozygosity and normal copy number of DNA segments which are not homozygous in the germ-line or normal somatic genome [36]. Due to a lack of change in the copy number, UPD remains undetected by karyotyping, FISH and aCGH. The CytoSure Haematological Cancer and SNP array (8x60k) can identify on one slide during the same experiment both copy number variations and SNP, which enables detection of corresponding LOH and UPD regions. A significant advantage of this method is also no need to use the corresponding control DNA from the same patient. In our analysis big LOH regions matched to deletion regions confirming presence of these changes by using another method. In most cases LOH corresponded to deletions with prognostic significance in CLL as 11q, 13q and 17p, what is in accordance with previous SNP array studies in CLL [24, 29]. UPD regions, showing changes in SNP distribution but not in copy number, were included in our analysis when covered regions bigger than 10 Mb [29]. In two patients with biallelic 13q14 deletion we have detected UPD regions. In one patient this neutral copy number LOH covered whole chromosome 13. In the second case small biallelic deletion was located in much bigger UPD region. Similar observation regarding the coexistence of UPD and biallelic 13q14 deletions was reported by other authors [16, 24, 29, 30]. The same size of deletion in both cases with UPD on chromosome 13 confirms duplication of deleted region, which is different from biallelic deletions with different sizes which probably arisen by two events. Biallelic 13q14 deletions of the same size but without copy neural LOH can be created by other genetic mechanism or the second deletion is invisible in array analysis because of to low percentage of clone with the second loss. UPD containing deletions may implicate the elimination of tumor suppressor genes. In one patient big UPD segment covered small gain region on 2p. In this case UPD is connected with gain of two oncogenes *REL* and *BCL11A* and hypothetically can concern unmutated gene copies or gene mutations increasing the activity of oncogenes. The significance of a common occurrence of UPD and copy number changes is not exactly defined, but can be related with clonal evolution favouring alleles with greater growth potential.

In the summary, the CytoSure Haematological Cancer and SNP array (8x60k) can precisely detect recurrent copy number changes with known prognostic significance in CLL/SLL as well as other chromosomal imbalances. The big advantage of this array is simultaneous detection of LOH and UPD regions during the same test. Resolution of this technique can accurately define size of 13q14 deletion with detection of miR-15a and miR-16-1 involvement. The average size of monoallelic 13q14 deletions was larger than in biallelic group. Our results show that bigger deletion including *RB1* or presence of biallelic 13q14 deletion is not sufficient to be considered as adverse prognostic factor. Uniparental disomies especially on chromosome 13 are quite frequent phenomenon in CLL patients, especially with biallelic 13q14 deletion and its impact on the disease course has to be determined.

## Methods

### Patients

The study group included 40 patients with diagnosis of CLL/SLL. All patients were evaluated in MSCM Institute and Cancer Center, Warsaw from February 2005 to November 2014. All samples had approval of the Bioethics Committee of the Oncology Centre - Institute Maria Sklodowska-Curie. The diagnosis of CLL/SLL was established between September 1999 and June 2014, according to the current WHO classification [29, 37]. For the present study patients were selected on the basis of the presence of 13q14 deletion detected by routine FISH analysis and the availability of specimens.

### Cell culture and cytogenetics

Fresh blood (CLL) or biopsy samples (SLL) were fixed directly or cultured in a 5 % CO2 atmosphere at 37 °C. The growth medium was DMEM (Lonza, Verviers, Belgium), enriched with 15 % fetal calf serum (GIBCO, Invitrogen GmbH, Karlsruhe, Germany) and antibiotics. Blood was cultured for 72 h and stimulated in two variants: with TPA (phorbol 12- myristate 13-acetate)(Sigma-Aldrich, Steinheim, Germany) or with DSP-30 (2 μM; TIBMolBiol, Berlin, Germany) together with IL-2 (200 U/ml; R&D Systems, Minneapolis, MN, USA). For biopsy material following cell cultures were performed: direct, 24 h without mitogens and 72 h with TPA or with DSP-30 plus IL-2. Cells for cytogenetic and FISH analysis were harvested according to standard procedures, cultures were treated with colcemid, afterwards cells were exposed to hypotonic solution and fixed in Cornoy’s solution. Chromosomes were Wright stained for G,C-banding. At least 7 metaphases were analyzed. Karyotypes were classified according to the International System for Human Cytogenetic Nomenclature (2013)[38].

### Fluorescence in situ hybridization (FISH)

FISH analysis was performed on tumor cells obtained directly from a biopsy or after unstimulated or stimulated in vitro culture. FISH was performed to establish the status of TP53, ATM, centromere12 and D13S319 region. Following commercially available probes were used: LSI TP53, LSI ATM, CEP12, LSI D13S319 and LSI 13q34 (Vysis Abbott Molecular, Downers, Grove, IL, USA). Loss of one D13S319 signal was equal with monoallelic 13q14 deletion and loss of both D13S319 signals was equivalent biallelic 13q14 deletion. The procedures for all commercial probes were applied according to the manufacturer’s protocol. At least 100 interphase cells were analysed. Slides were analyzed using an epifluorescence microscope Axioskop2 (Carl Zeiss, Jena, Germany) and documented by ISIS Imaging System (Metasysytems, Altlussheim, Germany).

### Array comparative genomic hybridization (aCGH)

DNA was extracted from fresh biopsy material or cytogenetic fixed cell suspension by QIAmp DNA Blood Mini Kit (Qiagen, Valencia, CA) according to the manufacturer’s recommendation. For aCGH analysis CytoSureTM Haematological Cancer and SNP Array (8x60k) (Oxford Gene Technology (OGT), Yarnton, Oxford OX5 1PF UK) was used. On this array average gene resolution was 68 Kb and SNP resolution was equal 30 Mb. The procedure of aCGH was performed following the manufacturer’s protocol. The reference DNA was from two pools of normal individuals (male and female), run as a same-sex control. Each patient and reference DNA was labeled with Cy3 and Cy5, respectively. Purification of labeled products, hybridization, and post-wash of the array was carried out according to OGT’s recommendation and with their proprietary solutions. Array slides were scanned with Agilent’s DNA Microarray Scanner and extraction software (Agilent, Santa Clara, USA).

### aCGH analysis

CytoSure Interpret software 020022 (OGT) was used for analysis of array data. The program uses the Circular Binary Segmentation (CBS) algorithm to generate segments along the chromosomes that have similar copy number relative to reference chromosome [39]. Averaging of the segments is with median value of all segments on a chromosome as the baseline. Deletion or duplication calls are made using the log2 ratio of each segment that has a minimum of four probes. Threshold factor for deletions was set as a log2 ratio of −0.6 that is less stringent than the theoretical log2 score of −1 (heterozygous deletion log2(1/2) = −1; No change in allele number log2(2/2) = 0; heterozygous duplication log2(3/2) = 0.59). The software uses the Derivative Log Ratio (DLR) Spread, which is used as a quality control check. This metric calculates probe-to-probe log ratio noise of an array and hence of the minimum log ratio difference required to make reliable amplification or deletion calls. A DLR of 0.08–0.19 is accepted, 0.20- 0.29 is borderline, and ≥0.30 is rejected. The DLR for all arrays was scored by this scale. Genes positions were identified according to human genome build hg19. The software calculated the total percentage homozygosity of each sample containing SNP data based on the method described by Sund et al.[40].

### PCR amplification of immunoglobulin rearrangements and sequence analysis

Genomic DNA was isolated from cell culture using the QIAamp DNA Extraction Kit (Qiagen, Hilden, Germany) according to the kit’s instructions. Immunoglobulin heavy chain variable gene (IGHV) rearrangements were amplified by the Multiplex polymerase chain reaction (PCR), following the BIOMED–2 protocol [41]. In this instance, each reaction contained a mixture of six family-specific framework region (FR) primers (VH1-VH6) and an antisense primer (JH). However, for cases, where mutations weren’t detected, *IGHV* rearrangement were determined by amplifying DNA using the appropriate leader primers. The cycling conditions were: an initial denaturation step at 95o C for 7 min, followed by 35 cycles at 94o C for 30 s, 60o C for 30 s and 72o C for 30 s, with a final extension step at 72o C for 7 min and ended at 4o C. The PCR products were determined by 2 % agarose gel electrophoresis. DNA bands were observed on the UV transilluminator and documented using the Bio-RAD software. PCR products were then purified using a mixture of two enzymes: alkaline phosphatase and exonuclease I (in the ratio 1:1). The purified amplicons were sequenced using the Big Dye Terminator and analysed with an automatic ABI PRISM 3100 Sequencer (Life Technology, Foster City, SA). Nucleotide sequences were analysed using the ImMunoGeneTics database (IMGT) [42]. Mutational status was identified by comparing the sequence of the IGHV of the patient with the most homologous germline V sequence. *IGHV* sequences with <98 % homology to a germline were defined as mutated, while sequences with of homology of 98 % or higher were considered as unmutated.

### Statistical methods

TTT was measured from the date of diagnosis until first treatment or, for untreated patients, to last follow-up (censored observation). OS was estimated from the date of diagnosis to the death (whatever the cause) or the last follow up. The cumulative probability of OS and TTT were plotted as curves according to the Kaplan-Meier method. A log-rank (Mantel-Cox) test was performed for all categorical variables. A *P*−value of <0.05 was considered as statistically significant.

## Abbreviations

CLL/SLL: Chronic lymphocytic leukemia/small lymphocytic lymphoma
FISH: Fluorescence in situ hybridization
aCGH: Array-comparative genomic hybridization
SNP: Single nucleotide polymorphism
LOH: Loss of heterozygosity
UPD: Uniparental disomy
*IGVH*: Immunoglobulin heavy-chain variable-region
MDR: Minimal deleted region
CNV: Copy number variations
M: Mutated *IGVH* status
UM: Unmutated *IGVH* status
TTT: Time to treatment
OS: Overall survival
TPA: Phorbol 12- myristate 13-acetate
DSP-30: CpG-oligonucleotide
IL-2: Interleukin 2
PCR: Polymerase chain reaction
IMGT: ImMunoGeneTics database
